# Prenatal Diagnosis of a Fetus with Congenital Heart Defect and Ring Chromosome 14

**DOI:** 10.1155/2012/794075

**Published:** 2012-11-05

**Authors:** Javier Sánchez, Lutgardo García-Díaz, David Chinchón, Guillermo Antiñolo

**Affiliations:** ^1^Unidad de Gestión Clínica de Genética, Reproducción y Medicina Fetal, Instituto de Biomedicina de Sevilla (IBIS), Hospital Universitario Virgen del Rocío (CSIC), Universidad de Sevilla, 41013 Seville, Spain; ^2^Unidad de Gestión Clínica de Anatomía Patológica, Hospital Universitario Virgen del Rocío, 41013 Seville, Spain; ^3^Centro de Investigación Biomédica en Red de Enfermedades Raras (CIBERER), 46010 Valencia, Spain

## Abstract

Monosomy of chromosome 14 has been reported in only a few prenatal cases. Generally, this monosomy is associated with a mosaicism of ring chromosome 14. Ring chromosome 14 is a rare cytogenetic entity with clinical characteristics that include growth retardation, facial dysmorphia, hypotonia, seizures, and retinitis pigmentosa. Given that the majority of symptoms appear postnatally, few cases have been reported of prenatal diagnosis of mosaicism monosomy/ring chromosome 14. We describe the prenatal diagnosis of a case of chromosomal mosaicism, a cell line with ring chromosome 14, r(14), and a second cell line with monosomy 14, in a fetus with aortic coarctation and chamber asymmetry. This is the first case of a prenatal diagnosis associating mosaicism with ring chromosome 14, monosomy 14, and fetal cardiopathy. We identified the exact breakpoint in ring chromosome 14 in IGH locus, which may provide further insight into the mode of ring formation as well as prenatal findings.

## 1. Introduction

Monosomy of chromosome 14 has been reported in only a few prenatal cases. Generally, this monosomy is associated with a mosaicism of ring chromosome 14. Ring chromosome 14 is a rare cytogenetic entity with clinical characteristics that include growth retardation, facial dysmorphia, hypotonia, seizures, and retinitis pigmentosa [1, 2]. Given that the majority of symptoms appear postnatally, few cases have been reported of prenatal diagnosis of mosaicism monosomy/ring chromosome 14 [3–5]. We describe the prenatal diagnosis of a case of chromosomal mosaicism, a cell line with ring chromosome 14, r(14), and a second cell line with monosomy 14, in a fetus with aortic coarctation and chamber asymmetry. This is the first case of a prenatal diagnosis associating mosaicism with ring chromosome 14, monosomy 14, and fetal cardiopathy.

## 2. Case Report

**Figure 1:**
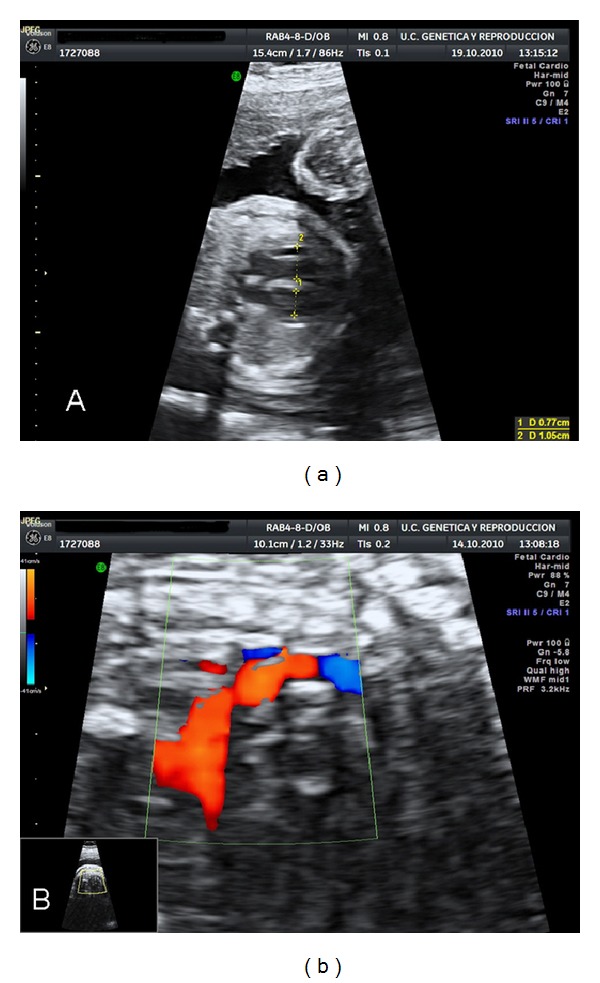
(a) Ultrasound showing chamber asymmetry. Lower: right ventricle (1.05 cm); upper: left ventricle (0.77 cm). (b) Aortic coarctation. Arrow: aortic isthmus.

A 32-year-old pregnant woman was admitted to our unit at 26 5/7 weeks of gestation to be assessed for possible fetal cardiopathy. Ultrasound revealed chamber asymmetry (right ventricle 0.77 cm versus left ventricle 1.05 cm) (Figure 1(a)), and aortic coarctation (Figure 1(b)). Foetal biometry did not reveal any other malformations; biparietal diameter was 61.5 mm (−2 SD); frontooccipital diameter was 85.7 mm (−0.5 SD); cranial circumference was 231.2 mm (−2 SD) and femur length was 49.0 mm (−0.25 SD). There was nothing of note in the family history; the parents were not consanguineous and this was the mother's first pregnancy. First trimester screening revealed low risk. 

Cordocentesis was performed using a 20 G needle under ultrasound guidance, obtaining 1 mL of fetal blood. Initially during this procedure, 20 mL of clear yellow amniotic fluid was obtained.

**Figure 2:**
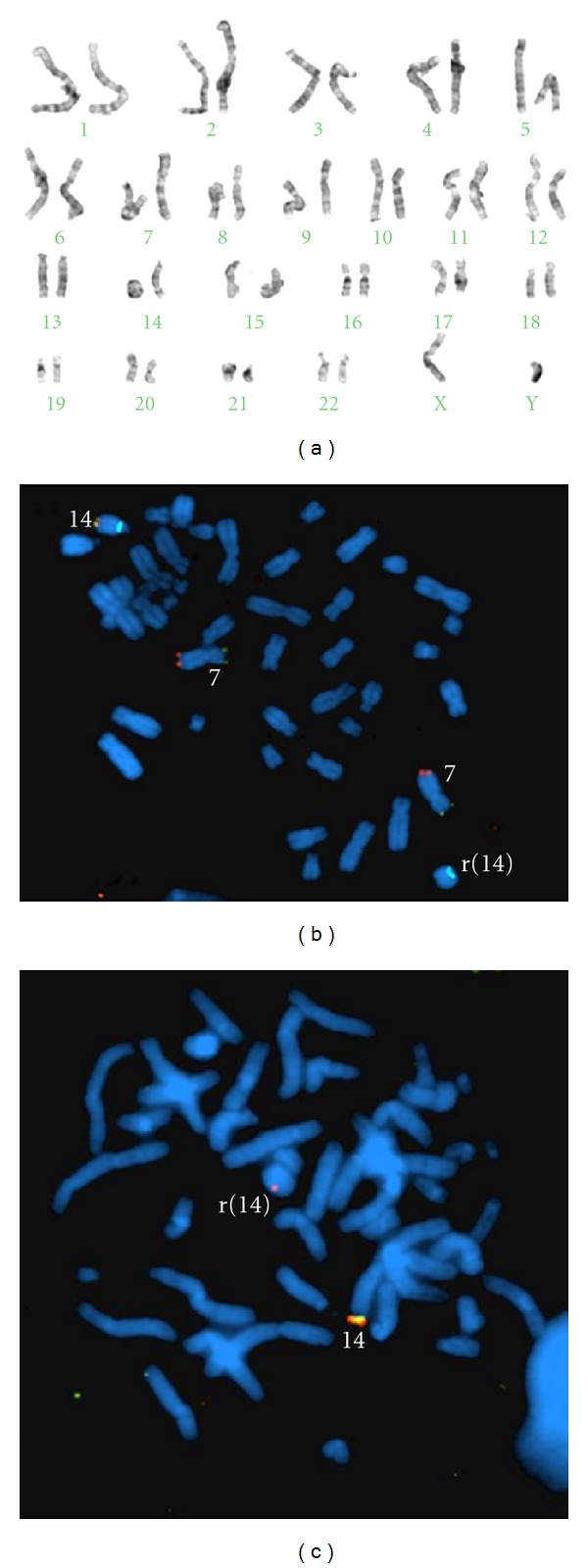
(a) Karyotype: 46,XY,r(14)(p11.2q32.33). (b) Hybridisation with ToTelVysion Mix 7 probes. Note the loss of signal corresponding to 14qter, maintaining the SpectrumAqua 14q control signal. (c) In situ hybridisation with LSI IGH Dual Color, Break Apart Rearrangement Probe. Note the two hybridisation signals in the normal chromosome 14 (red/green signal). Loss of LSI IGHV Probe (SpectrumGreen) was detected in chromosome r(14).

The analysis, using FISH with the AneuVYsion kit (Vysis Downers Grove, IL, USA) of uncultured amniotic fluid cells was compatible with a male fetus with a normal complement of the chromosomes studied (X, Y, 13, 18, and 21). A cytogenetic study of in fetal lymphocytes stimulated after 72-hour culture, revealed the presence of two cell lines: a major cell line with 46 chromosomes in which a ring chromosome was identified (Figure 2(a)), and a second cell line with 45 chromosomes, with a monosomy of chromosome 14, chromosome formula (ISCN 2009): 45,XY,-14[3]/46,XY,r(14)(p11.2q32.33)[27]. The r(14) was characterized by fluorescent in situ hybridization (FISH) with mix 7 of the ToTelVysion kit (Vysis Downers Grove, IL, USA), which includes the 14qter subtelomeric specific probe, (D14S1420) and a 14q control probe (Figure 2(b)). For better characterization, we used the LSI IGH Dual Color, Break Apart Rearrangement Probe (Vysis Downers Grove, IL, USA). This kit consists of a combination of two probes that hybridize in the immunoglobulin heavy-chain locus: a proximal LSI IGH 3′ Flanking Probe (SpectrumOrange) and a distal LSI IGHV Probe (SpectrumGreen). These probes hybridize both sides of the J segment of the constant region of the IGH locus in such a way that any breakpoint located in the J segment will cause the separation or loss of one of the probes (Abbott molecular: probes for FISH analysis; http://www.abbottmolecular.com/). FISH analysis with a subtelomeric probe showed deletion of the 14qter subtelomeric region in the ring chromosome, while there was a 14 q control probe signal. The study with LSI IGH probes showed that there was loss of the distal LSI IGHV probe in the r(14), located at the breakpoint between the two probes. Consequently, we estimated that the r(14) had a loss of 260 kb (UCSC Genome Browser database; http://genome.ucsc.edu/). Cytogenetic analysis of cultured amniotic fluid cells confirmed the presence of both cell lines. The percentages of cell lines, however, were different from those found in foetal lymphocytes, with the most frequent observed corresponding to one reflecting monosomy 14: 45,XY,-14[19]/46,XY,r(14)(p11.2q32.22)[5]. Therefore, the fetal karyotype is 45,XY,-14/46,XY,r(14)(p11.2q32.33).ish r(14)(5′IGH-,3′IGHx1,D14S1420-). While 90% of the fetal lymphocyte cells showed the ring chromosome, only 21% of the amniotic fluid cells showed the ring chromosome. In this sample, the most frequent cell line observed corresponds to monosomy 14. Cytogenetic analysis in both parents was normal.

Given the cytogenetic results, the parents decided to terminate the pregnancy at week 27 5/7 of gestation. Autopsy revealed a male fetus weighing 1038 g (90 percentile) with a length of 40.5 cm (50 percentile) and facial dysmorphia. The heart, weighing 7 g (50 percentile), showed a prominent right ventricle and pulmonary artery, and an aortic arch with stenosis of the descending aorta. The position and structure of the other organs were within the normal range. The placenta weighed 249 g (50 percentile) and had a single umbilical artery. Microscopic analysis of the organs was normal.

## 3. Discussion

Few cases have been reported of prenatal monosomy mosaicism and ring chromosome 14. Jean et al. reported a case with cystic hygroma of 8 mm after ICSI [3] and more recently Quenum-Miraillet et al. reported a fetus with severe skeletal dysplasia [5]. Neither report mentioned fetal cardiac abnormalities. Most cases of mosaicism with r(14) diagnosed postnatally do not present relevant prenatal history. Clinical manifestations include characteristic facies, hypotonia, postnatal onset microcephaly, and retinitis pigmentosa. Patients with r(14) present with drug-resistant seizures, which tend to disappear during adolescence. There are no major malformations, however, and most have postnatal onset, which hinders their prenatal identification [2]. The phenotypic differences observed in these patients may be caused by the actual somatic mosaicism due to the mitotic instability of the ring chromosome. This tissue-specific instability was detected in our case. While most of the cells in the 72-hour culture of fetal lymphocytes showed the ring chromosome, the presence of the ring chromosome in amniotic fluid cells after 10–12 days of culture was only 21%. It seems, therefore, that the phenotype associated with this entity is due not only to the loss of genetic material and therefore the size of the ring, but also to the mitotic instability of the tissue-specific ring chromosome. It could also be due to the possible gain of material observed in some cases, a process called “dynamic tissue-specific mosaicism” [6]. In both patients with ring chromosomes and in those with deletions of the 14q22.1q22.1 region, one of the symptoms observed repeatedly is susceptibility to infections, which may even cause death. In our case, we have been able to identify the breakpoint, located distally at the LSI IGH 3′ probe in the J-segment of the immunoglobulin gene. As a result, the immunoglobulin heavy chain gene is disrupted and consequently presents haploinsufficiency in this gene. This may account for the recurrent respiratory infections observed in these patients [4]. Moreover, the formation of the ring chromosome alters the chromosomal structure modulating the expression of genes, which under normal conditions would be very distant. Therefore, patients with the same breakpoint present phenotypic variations [7]. In our case, the regulatory regions of the IGH gene may be located in areas that influence genes that under normal conditions would be very distant [8]. This is the first case of a prenatal diagnosis of monosomy mosaicism/ring chromosome with fetal cardiopathy. Given that the majority of symptoms in patients with ring chromosome 14 have postnatal onset, prenatal diagnosis of this entity has only been reported in a few cases. In our case, the presence of fetal cardiopathy leads us to identify the chromosomal anomaly. Various studies have performed genotype-phenotype correlations in patients with terminal 14q32.33 deletion [2, 9, 10]. The presence of congenital cardiopathy, however, has only been described in one patient [11]. 

The onset of dynamic tissue-specific mosaicism in fetuses with ring chromosome 14 makes the interpretation of cytogenetic prenatal diagnosis difficult, especially because of the absence of major fetal malformations. Mitotic instability of r(14) may lead to errors in diagnosis if a sufficient number of cells are not analyzed, or if they are not present in the fetal tissue studied. In cases in which cytogenetic analysis reveals the presence of chromosome 14 monosomy, the presence of an r(14) should be suspected over a pseudomosaicism. In these cases, and when faced with a lack of ultrasound abnormalities, a fetal lymphocyte study provides valuable information for interpreting the fetal karyotype. 

An informed consent was obtained from all the participants for clinical and molecular genetic studies. The study conformed to the tenets of the declaration of Helsinki as well as the requirements established by our institutional review board.

## References

[1] Fryns JP, Kubien E, Kleczkowska A (1983). Ring chromosome 14. A distinct clinical entity. *Journal de Génétique Humaine*.

[2] Zollino M, Seminara L, Orteschi D (2009). The ring 14 syndrome: clinical and molecular definition. *American Journal of Medical Genetics A*.

[3] Jean M, Rival JM, Mensier A, Mirallié S, Lopes P, Barrière P (1997). Prenatal diagnosis of ring chromosome 14 after intracytoplasmic sperm injection. *Fertility and Sterility*.

[4] McConnell V, Derham R, McManus D, Morrison PJ (2004). Mosaic monosomy 14: clinical features and recognizable facies. *Clinical Dysmorphology*.

[5] Quenum-Miraillet G, Malan V, Martinovic J (2008). Prenatal diagnosis of a ring chromosome 14 in a fetus with a severe skeletal dysplasia. *Prenatal Diagnosis*.

[6] Sodré CP, Guilherme RS, Meloni VFA (2010). Ring chromosome instability evaluation in six patients with autosomal rings. *Genetics and Molecular Research*.

[7] Klein CB, Costa M (1997). DNA methylation, heterochromatin and epigenetic carcinogens. *Mutation Research*.

[8] Castermans D, Thienpont B, Volders K (2008). Position effect leading to haploinsufficiency in a mosaic ring chromosome 14 in a boy with autism. *European Journal of Human Genetics*.

[9] Maurin ML, Brisset S, Le Lorc’h M (2006). Terminal 14q32.33 deletion: genotype-phenotype correlation. *American Journal of Medical Genetics*.

[10] Youngs EL, Hellings JA, Butler MG (2011). A clinical report and further delineation of the 14q32 deletion syndrome. *Clinical Dysmorphology*.

[11] Meschede D, Exeler R, Wittwer B, Horst J (1998). Submicroscopic deletion in 14q32.3 through a de novo tandem translocation between 14q and 21p. *American Journal of Medical Genetics*.

